# Global Gene Expression Profiling Reveals Functional Importance of Sirt2 in Endothelial Cells under Oxidative Stress

**DOI:** 10.3390/ijms14035633

**Published:** 2013-03-11

**Authors:** Junni Liu, Xiao Wu, Xi Wang, Yun Zhang, Peili Bu, Qunye Zhang, Fan Jiang

**Affiliations:** 1 Key Laboratory of Cardiovascular Remodeling and Function Research, Chinese Ministry of Education and Chinese Ministry of Public Health, Shandong University, Jinan 250012, Shandong, China; E-Mails: liujunni2012sdu@163.com (J.L.); wuxiao1022@126.com (X.Wu.); wang_xi@126.com (X.Wa.); zhangyun@sdu.edu.cn (Y.Z.); 2 Department of Cardiology, Qilu Hospital, Shandong University, Jinan 250012, Shandong, China

**Keywords:** Sirt1, Sirt2, endothelial cell, oxidative stress, functional genomics, microarray

## Abstract

The NAD^+^-dependent deacetylases Sirt1 and Sirt2 mediate cellular stress responses and are highly expressed in vascular endothelial cells. In contrast to the well-documented protective actions of Sirt1, the role of endothelial Sirt2 remains unknown. Using cDNA microarray and PCR validation, we examined global gene expression changes in response to Sirt2 knock down in primary human umbilical vein endothelial cells under oxidative stress. We found that Sirt2 knock down changed expression of 340 genes, which are mainly involved in cellular processes including actin binding, cellular amino acid metabolic process, transmembrane receptor protein serine/threonine kinase signaling, ferrous iron transport, protein transport and localization, cell morphogenesis, and functions associated with endosome membrane and the trans-Golgi network. These genes and associated functions were largely non-overlapping with those altered by Sirt1 knock down. Moreover, we showed that pharmacological inhibition of Sirt2 attenuated oxidant-induced cell toxicity in endothelial cells. These suggest that Sirt2 is functionally important in endothelial cells under oxidative stress, and may have a primarily distinct role as compared to Sirt1. Our results may provide a basis for future studies aiming to dissect the specific signaling pathway(s) that mediates specific Sirt2 functions in endothelial cells.

Abbreviations

eNOS: nitric oxide synthase
NF-κB: nuclear factor-κB
HUVEC: human umbilical vein endothelial cell
siRNA: small interfering RNA
qPCR: quantitative polymerase chain reaction
GO: Gene Ontology
GPx: glutathione peroxidase
SOD: superoxide dismutase

## 1. Introduction

Mammalian Sirt proteins (Sirt1 to Sirt7) are orthologues of the yeast *SIR2* gene product, an NAD^+^-dependent class III histone deacetylase [1–4]. All Sirt proteins contain a conserved NAD^+^-dependent catalytic core domain of ~275 amino acids [3,4]. Among the seven Sirt proteins identified, Sirt1, 2, and 3 have the highest homology to yeast Sir2 and all exhibit specific protein deacetylase activity [5,6]. In addition to their important roles in aging and metabolic regulation, different studies have suggested that Sirt is also involved in modulating cardiovascular physiology and disease [1,7]. In the heart, for example, Sirt1 and Sirt3 have been implicated in promoting cardiomyocyte survival and preventing cardiac remodeling in response to different stress stimuli [8]. In blood vessels, activation of Sirt functions, especially those of Sirt1, is associated with multiple beneficial effects such as preventing vascular cell senescence, suppressing inflammation, decreasing cellular oxidative stress, and promoting vascular regeneration [1,7]. Moreover, Sirt may also exert cardiovascular protective actions by improving global glucose and lipid metabolism [8].

Endothelial cells have a pivotal role in maintaining the homeostasis of blood vessels. Endothelial dysfunction is recognized as a major cellular basis of the development of many cardiovascular diseases such as hypertension, atherosclerosis and heart failure [9]. Several lines of evidence have indicated that Sirt1 has an important role in modulating endothelial cell functions. In particular, Sirt1 physically interacts with and deacetylates endothelial nitric oxide synthase (eNOS), leading to enhanced eNOS activation [10]. Both *in vitro* and *in vivo* experiments revealed that activation of Sirt1 function led to increases in nitric oxide production and endothelium-dependent vasorelaxation, decreased inflammatory reactions in endothelial cells, and suppressed endothelial cell apoptosis and senescence [1,11–14].

Several Sirt members have pivotal roles in modulating cellular stress responses [2,6]. Under oxidative stress, Sirt1 exhibited broad cytoprotective effects in endothelial cells [7,15–19]. The molecular mechanisms by which Sirt1 produces these cytoprotective actions are not totally understood, while current evidence indicates that activation of the FoxO family members by Sirt1 is likely to be a major signaling route [2,6]. In contrast to Sirt1, the biological functions of Sirt2 in endothelial cells remain unknown [1]. Results from previous studies in non-endothelial cells indicate that the effects of Sirt2 on cell viability under stress are variable and appear to be cell type- and context-dependent [20–27]. Currently, research efforts have been made in the development of selective Sirt2 inhibitors, which may be used as novel chemotherapy agents [28]. Hence, it is important to determine whether and how Sirt2 is involved in modulating endothelial cell homeostasis under stress conditions. Like Sirt1, Sirt2 expression is also responsive to oxidative stress [24]. Moreover, Sirt2 and Sirt1 share a number of common substrates, including FoxO1, FoxO3, nuclear factor (NF)-κB, histone H3 and p300 [4,8,24,29–32]. These results prompted us to hypothesize that Sirt2 may also have critical functions in endothelial cells under oxidative stress. Therefore, in the present study we aim to examine the importance of endothelial Sirt2 on a systematic biology basis, by characterizing global gene expression changes after Sirt2 knock down in primary human umbilical vein endothelial cells (HUVECs) using mRNA microarray, an approach that has been used to delineate Sirt1 functions at the whole genome level [33,34].

## 2. Results and Discussion

**Figure 1:**
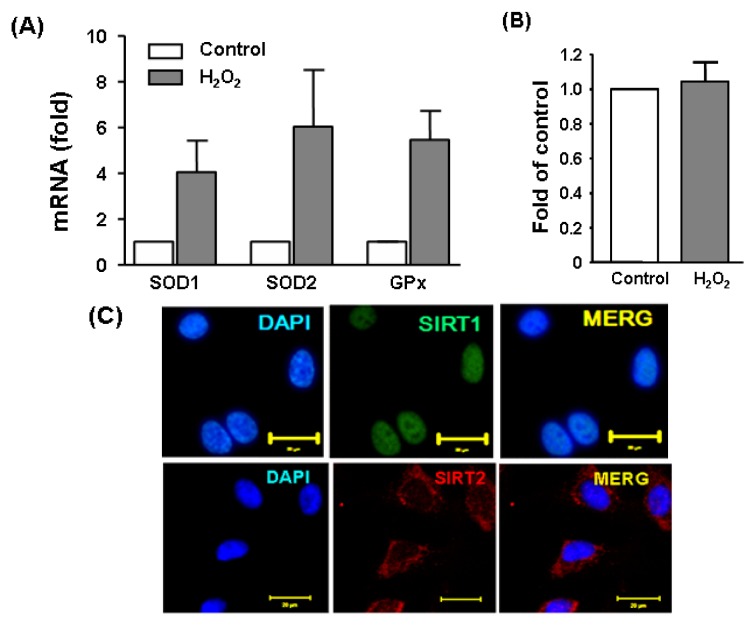
(**A**) An oxidative stress response induced by H_2_O_2_ (300 μM for 6 h) in cultured human umbilical vein endothelial cells (HUVECs), as revealed by the upregulation of glutathione peroxidase (GPx) and superoxide dismutases (SOD) as measured by qPCR; (**B**) H_2_O_2_ treatment at 300 μM did not result in obvious cytotoxicity in the present experimental system. Cell viability was assessed with a MTS-based assay. Data are mean ± SEM, *n* = 3–4; (**C**) pseudo-colored immunofluorescence images showing the intracellular localization of Sirt1 and Sirt2 in untreated HUVEC. Nuclei were counter stained with DAPI (blue). Bar = 20 μm. MERG, merged image.

To induce oxidative stress in cultured endothelial cells, we treated the cells with H_2_O_2_ at 300 μM for 6 h. Induction of a cellular stress response under this condition was demonstrated by the upregulation of detoxification enzymes glutathione peroxidase (GPx) and superoxide dismutases (SOD) as measured by qPCR (Figure 1A). We also confirmed that this treatment protocol did not induce obvious cytotoxic effects as assessed by a MTS cell viability assay (Figure 1B). A previous study demonstrated that Sirt2 expression was upregulated upon oxidant stimulation in adipocytes [24]. To clarify whether this is also the case in endothelial cells, we measured Sirt2 expression with qPCR in H_2_O_2_ challenged cells. We found that as compared to untreated cells, H_2_O_2_ increased Sirt2 expression by ~2 fold (data not shown). To demonstrate the intracellular localization of Sirt2 and in endothelial cells, we performed immunofluorescence staining. As shown in Figure 1C, the majority of Sirt2 showed a cytosolic localization, which was in contrast to Sirt1, which was mainly nuclear.

**Figure 2:**
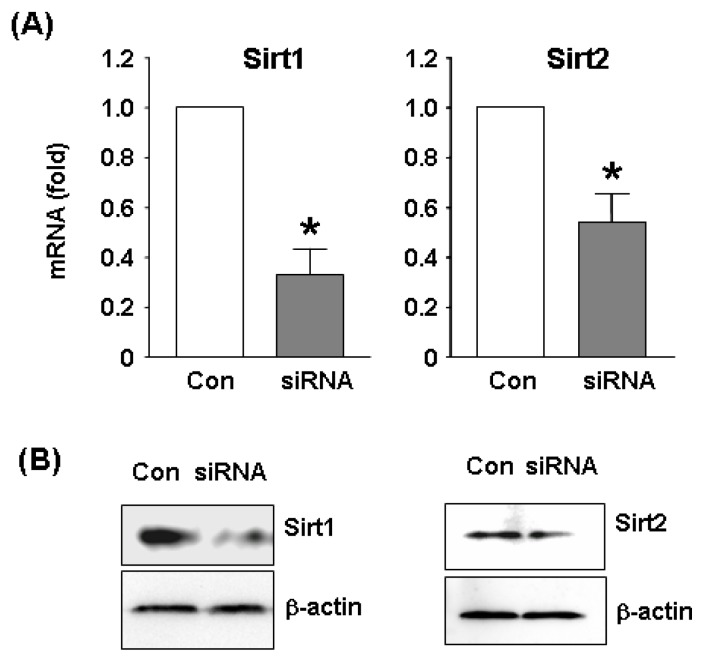
(**A**) qPCR and (**B**) Western blot results showing gene silencing efficiency of siRNA sequences targeting Sirt1 or Sirt2 in H_2_O_2_-treated HUVECs. A non-specific siRNA was used as control. Data are mean ± SEM. * *p* < 0.05, student’s *t*-test, *n* = 4–5. Western blots were representative images from two independent experiments.

**Figure 3:**
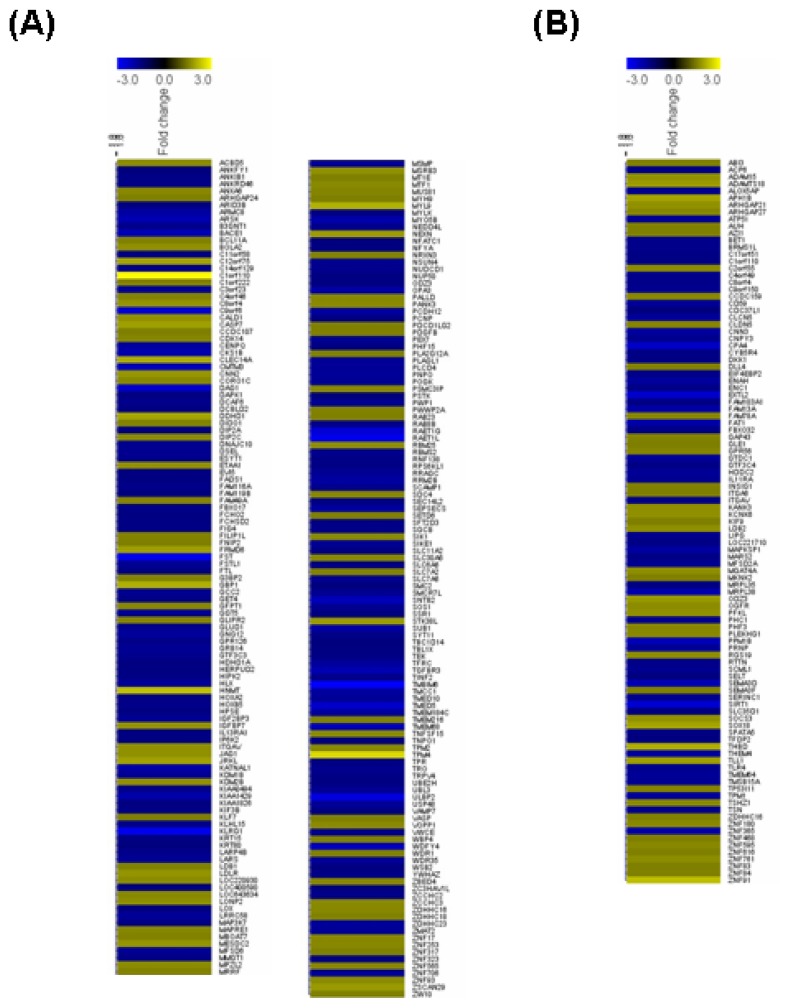

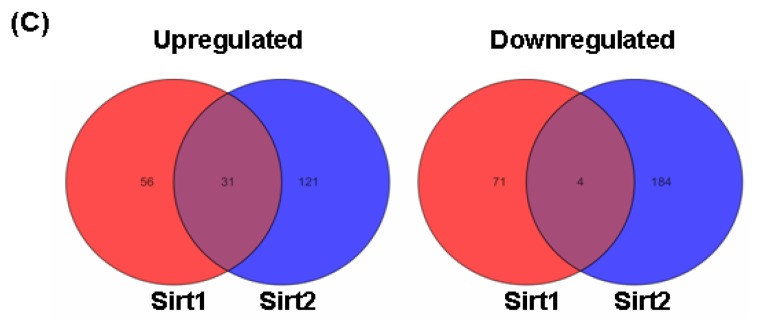
Heat map diagrams illustrating the significantly changed (*p* < 0.05 with a fold change value >1.5 as compared to control cells) genes in HUVECs with gene silencing of (**A**) Sirt2 and (**B**) Sirt1, determined by Affymetrix Human Genome U219 Array (*n* = 3 biological replicates each); (**C**) Venn graphs showing the number of genes up- and downregulated by Sirt1 or Sirt2 gene silencing. A high-resolution version for Figure 3A,B is available online.

To clarify the functions of Sirt2 in endothelial cells at the genome level, we performed microarray experiments comparing the global gene expression profiles between control and Sirt2i cells. The efficiency of siRNA-mediated gene knock down was confirmed by qPCR and Western blot (Figure 2). We showed that under oxidative stress conditions, knock down of Sirt2 significantly changed the expression level of 340 genes, with 152 being upregulated and 188 downregulated (Figure 3A and Table 1). GO analysis of the Sirt2-sensitive genes showed that these genes were mainly involved in cellular processes related to actin binding, cellular amino acid metabolic process, transmembrane receptor protein serine/threonine kinase signaling pathways, ferrous iron transport, protein transport and localization, cell morphogenesis involved in differentiation, and functions associated with endosome membrane and the trans-Golgi network.

**Figure 4:**
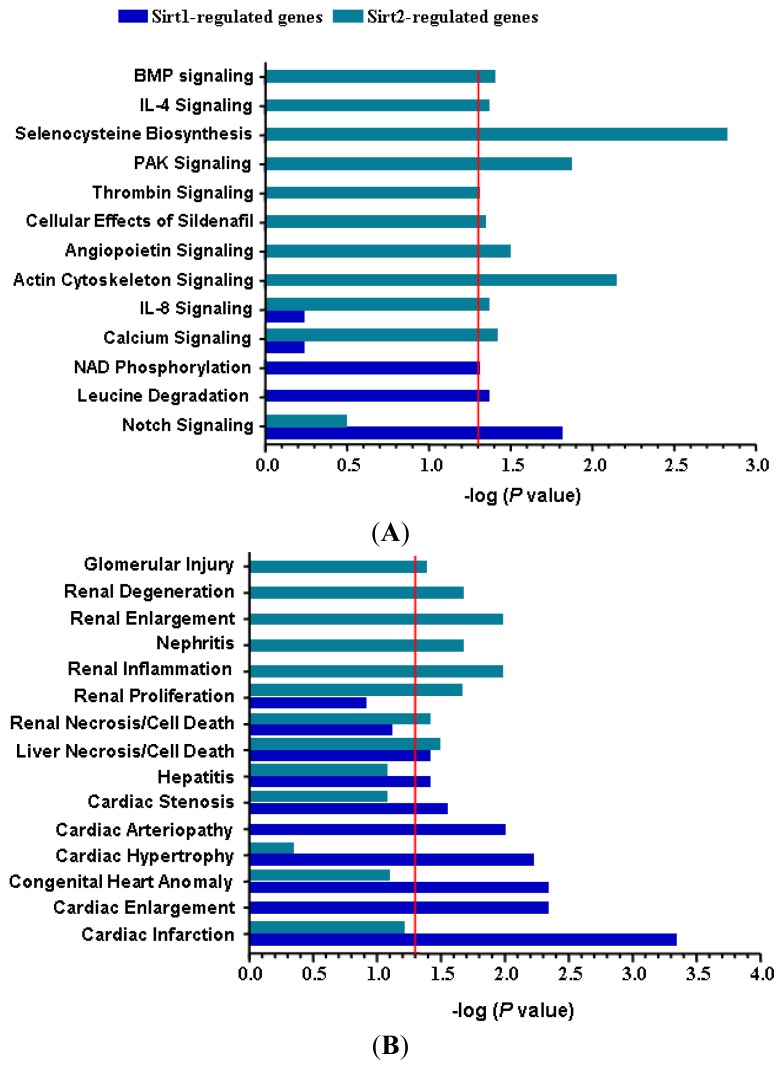
Comparison of the potential categories of (**A**) intracellular pathways and (**B**) biological mechanisms related to toxicity responses that were significantly over-represented by Sirt1- or Sirt2-regulated genes respectively in stressed endothelial cells. The red line indicates the threshold of statistical significance. Functional gene enrichment analysis was performed with IPA software.

To confirm that the altered gene expression after Sirt2 knock down was not caused by non-specific off target effects, we run a parallel experiment by knocking down Sirt1 using the same protocol. Sirt1 gene knock down induced significant alterations of expression of 162 genes (87 upregulated and 75 downregulated) (Figure 3B and Table 1). Among the upregulated genes with Sirt1i, only 31 (36%) overlapped with those changed in Sirt2i cells (20% of those changed in Sirt2i cells). Similarly, among the downregulated genes, only 4 (5%) overlapped with those changed in Sirt2i cells (2% of those changed in Sirt2i cells) (Figure 3C). GO analysis of the Sirt1-sensitive genes showed that these genes were mainly involved in cellular processes related to actin binding, ion binding, endoplasmic reticulum, cellular macromolecule biosynthetic process, cytoskeletal protein binding, and Golgi apparatus, of which the majority was distinct from those related to Sirt2. Further analysis of the differentially expressed genes in relation to disease processes with IPA software revealed that Sirt2-sensitive genes were enriched in categories including infectious disease, connective tissue disorders, developmental disorder, skeletal and muscular disorders, and cardiovascular disease. In comparison, Sirt1-sensitive genes were mainly enriched in categories including cardiovascular disease, inflammatory response, cancer, organismal injury and abnormalities, and connective tissue disorders. We also compared the two sets of cellular pathways significantly over-represented by Sirt1- or Sirt2-sensitive genes respectively, and found that the pathways affected by Sirt1 were primarily distinct from those affected by Sirt2 (Figure 4A). Moreover, IPA-Tox analysis revealed that Sirt1i and Sirt2i exhibited a discrete pattern of gene enrichment in categories of biological mechanisms that were related to toxicity responses (Figure 4B).

**Figure 5:**
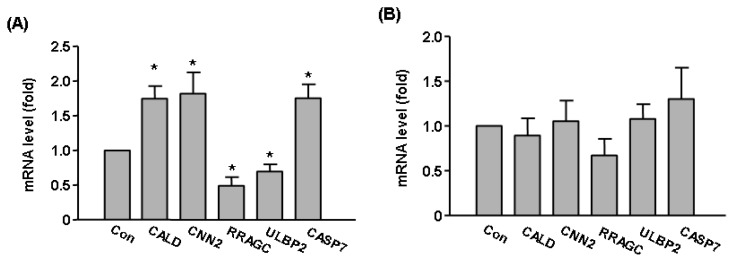
Validation of microarray results with qPCR. The expression levels of *CALD1*, *CASP7*, *CNN2*, *RRAGC*, *ULBP2* were measured in (**A**) Sirt2i cells and (**B**) Sirt1i cells in the presence of oxidant stress (H_2_O_2_ 300 μM for 6 h). Gene expression levels were expressed as fold of control. Data are mean ± SEM. *p < 0.05 *vs.* Con, Student’s *t*-test, *n* = 3–4.

**Figure 6:**
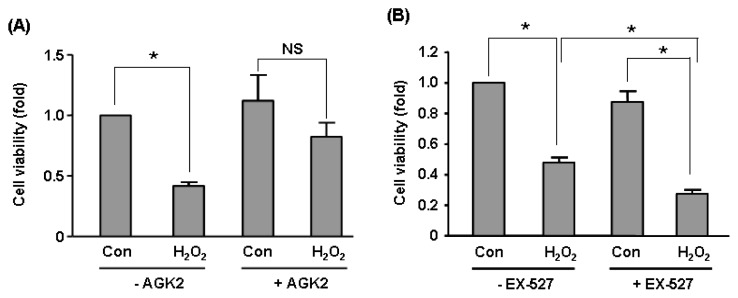
Effects of (**A**) selective Sirt2 inhibitor AGK2 (10 μM) and (**B**) selective Sirt1 inhibitor EX-527 (10 μM) on H_2_O_2_-induced cell toxicity in HUVECs measured with MTS assay. Cells were treated with H_2_O_2_ (600 μM) for 2 h in the presence and absence of AGK2 or EX-527 pre-treatment. Data are mean ± SEM. *p < 0.05, one-way ANOVA, *n* = 4–6. NS: non-significant.

To validate our microarray data of Sirt2 effects on global gene expression, we carried out qPCR assays on selected genes including *CALD1*, *CASP7*, *CNN2*, *RRAGC*, *ULBP2*. We showed that Sirt2i induced upregulation *CALD1*, *CASP7*, *CNN2* and downregulation of *RRAGC*, *ULBP2* (Figure 5A). These changes were in accordance with the trend as detected by microarray (see Table 1). In contrast, expressions of these genes were not altered in Sirt1i cells (Figure 5B). To further clarify whether Sirt2 was functionally important in endothelial cells under stress, we treated HUVEC cells with a higher concentration (600 μM) of H_2_O_2_ for 2 h in the absence and presence of a selective Sirt2 inhibitor AGK2 (from Merck, Darmstadt, Germany) [35]. We found that pre-treatment with AGK2 (10 μM) attenuated H_2_O_2_-induced cell toxicity (Figure 6A), suggesting that under oxidative stress, activation of the Sirt2 pathway might have a detrimental effect on cell viability. In contrast, we showed that pre-treatment with the selective Sirt1 inhibitor EX-527 (10 μM) (from Merck) increased H_2_O_2_-induced cell toxicity (Figure 6B).

In the present study, we explored the functional importance of Sirt2 in endothelial cells under oxidative stress by measuring global gene expression changes in cells in which Sirt2 was knocked down. We found that Sirt2 gene knock down significantly altered the expression profile of 340 genes, which were involved in different cellular processes (see Table 1). We also confirmed the microarray data with qPCR for selected genes. Gene clustering analysis suggests that Sirt2-sensitive genes in endothelial cells may be involved in regulation of protein transport and localization, cellular amino acid metabolic process, and functions associated with endosome membrane and the trans-Golgi network. These functional annotations are in agreement with findings from cellular function studies showing that Sirt2 may have a pivotal role in modulating cell autophagy, an intracellular mechanism responsible for clearance of damaged proteins and organelles involving activation and mobilization of the endogenous membranous system [36]. Interestingly, a recent study demonstrated that overexpression of Sirt2 inhibited lysosome-mediated autophagic turnover and increased the sensitivity of cells to proteasomal stress-induced cytotoxicity [37]. Conversely, accumulation of ubiquitinated proteins and cytotoxicity in stressed cells were attenuated by Sirt2 knock down. These results indicate that a complex interaction between Sirt2 and autophagic process may be present. In line with these findings, we observed that pharmacological inhibition of autophagy in endothelial cells augmented H_2_O_2_-induced cell death [38]. Moreover, we found that inhibition of Sirt2 decreased H_2_O_2_-induced endothelial cytotoxicity (see below). Taken together, we propose that regulation of cellular autophagic processes might be a mechanistic link between Sirt2 and oxidative stress-induced injury in endothelial cells. In addition to the above-mentioned pathways, results from our gene function clustering analysis indicate that Sirt2-regularted genes may also be involved in actin binding, transmembrane receptor protein serine/threonine kinase signaling pathways, ferrous iron transport, and cell morphogenesis involved in differentiation.

Similar to Sirt1, Sirt2 has strong deacetylase activity, and may affect gene expression by modulating functions of multiple transcription factors and co-activators such as FoxO, NF-κB, p300, and histone [4–6,23,24,29–32]. However, the present gene profiling study showed that the potential intracellular pathways regulated by Sirt2 in stressed endothelial cells were primarily different from those regulated by Sirt1. Consistently, Sirt1- and Sirt2-sensitive genes were involved in distinct categories of diseases, for example inflammatory response, cancer, and organismal injury for Sirt2, and infectious disease, developmental disorder, skeletal and muscular disorders for Sirt1. As observed in neural cells [39], our data suggest that Sirt2 is likely to have a distinct functional role from Sirt1 in endothelial cells under stress conditions. Moreover, these data support that the observed gene expression changes in response to Sirt2 knock down are unlikely to be a result of non-specific off target effects of RNA interference.

The precise cellular functions of Sirt2 in endothelial cells remain largely unknown. A previous study has shown that Sirt2 may be implicated in mediating angiotensin II-induced endothelial cell migration via modulating α-tubulin acetylation and microtubule reorganization [40]. Consistent with this observation, we identified (and confirmed with qPCR) that Sirt2 knock down altered the expression of several genes involved in cytoskeletal organization, cell contraction and migration, such as *CALD1* (caldesmon) and *CNN2* (calponin) [41,42]. Moreover, we demonstrated that Sirt2 also affected expression of genes involved in modulating cell viability. This is exemplified by *CASP7* (caspase 7), which is a master regulator of cell apoptosis, and *RRAGC* (Ras-related GTP binding C), which is involved in activation of the mTOR pathway [43].

To clarify the general role of Sirt2 in endothelial cells under oxidative stress, we challenged the cells with a high concentration of H_2_O_2_ and demonstrated that pharmacological inhibition of Sirt2 activity attenuated H_2_O_2_-induced cytotoxicity. This result is consistent with previous experiments in neural and cardiac cells showing that activation of Sirt2 promotes cell death, whereas knock down or inhibition of Sirt2 enhances cellular stress-tolerance [25,35]. Moreover, we confirmed that inhibitions of Sirt2 and Sirt1 had divergent effects on endothelial cell viability under H_2_O_2_-induced oxidative stress, an observation that was consistent with our microarray data revealing that there was only a small intersection between Sirt2- and Sirt1-sensitive genes in H_2_O_2_-challenged endothelial cells. Our experiments supported previous findings that Sirt1 exhibited profound cytoprotective effects in vascular endothelial cells in response to oxidative stress [7,15,16].

## 3. Experimental Section

### 3.1. Cell Culture

HUVECs were purchased from the American Type Culture Collection and maintained in ECM (from ScienCell Research Labortories, Carlsbad, CA, USA), supplemented with 10% fetal bovine serum, 1% ECG (endothelial cell growth supplement, ScienCell), and antibiotics (penicillin 100 U/mL, streptomycin 100 μg/mL). Cells were cultured at 37 °C with 5% CO_2_. Confluent cells were subcultured with 0.25% trypsin-EDTA, and cells of passage 3 to 5 were used for experimentation. Cell viability was assessed with the tetrazolium-based (MTS) assay using CellTiter 96 Aqueous kit (from Promega, Madison, WI, USA) according to the manufacturer’s direction.

### 3.2. RNA Interference

Small interfering RNA (siRNA) molecules targeting Sirt1 and Sirt2 were synthesized by GenePharma (Shanghai, China). For each target, 3 different siRNA sequences were tested with quantitative polymerase chain reaction (qPCR), and the one with highest efficacy was selected for following experiments. For siRNA transfection, cells were subcultured 24 h before treatment. Cells were incubated with siRNA (final concentration 30 nM) mixed with Lipofectamin RNAiMAX Reagent (Life Technologies, Carlsbad, CA, USA) for 6 h in antibiotic-free medium, and then changed to normal medium for additional 18 h.

### 3.3. Microarray Experiments and Data Processing

Cells were transfected with a control siRNA, Sirt2-specific siRNA (Sirt2i) or Sirt1-specific siRNA (Sirt1i). Three biological replicates were included for each group (hence a total of 9 arrays were analyzed). To induce oxidative stress, all transfected cells were treated with H_2_O_2_ at 300 μM for 6 h. Total RNA was isolated using TRIzol reagent (Life Technologies, Carlsbad, CA, USA) according to the manufacturer’s protocol. RNA quality was tested with Bioanalyzer 2100 (Agilent, Santa Clara, CA, USA) and further purified with RNeasy Micro kit (Qiagen, Hilden, Germany). Microarray analysis was performed using Affymetrix Human Genome U219 Array, using standard labeling, hybridization and scanning protocols (ShanghaiBio Corporation, China). The raw data were processed and analyzed with GeneSpring GX software. Genes with a fold change of >1.5 and with a *p* value of <0.05 as compared to control were selected as differentially expressed genes. Gene Ontology (GO) functional annotation of the differentially regulated genes was carried out using DAVID Bioinformatics Resources 6.7 [44]. Further gene function clustering analysis was performed with IPA software (Ingenuity Systems, Redwood City, CA, USA).

### 3.4. Real-Time qPCR

Total RNA (500 ng) was reverse transcribed to cDNA using Prime Script RT reagent Kit (TaKaRa Biotechnology, Dalian, China). Real-time qPCR was performed with TaqMan gene expression assays primer-probe sets (Applied Biosystems, Carlsbad, CA, USA) or using a Sybr green-based master mix kit (SsoFast EvaGreen from Bio-Rad, Hercules, CA, USA) according to the manufacturer’s instructions. *GAPDH* or *18S* was used as the housekeeping gene.

### 3.5. Fluorescent Immunocytochemistry

Cells grown on Lab-Tek II chamber slides (Nunc, Roskilde, Denmark) were fixed with cold methanol for 30 min, washed in PBS and blocked with 5% bovine serum albumin. Cells were incubated overnight with polyclonal anti-Sirt1 (1:200) (from Abcam, Cambridge, UK) or anti-Sirt2 (1:100) (from Millipore, Billerica, MA, USA). Immunofluorescent labeling was performed with DyLight594-conjugated donkey anti-rabbit IgG (1:400) (Jackson ImmunoResearch, West Grove, PA, USA). Cell nuclei were counter stained with DAPI. Images were captured using a Zeiss laser scanning confocal microscope (Zeiss LSM710, Oberkochen, Germany). Negative control experiments were performed using corresponding non-immune IgGs.

### 3.6. Western Blot

Total protein was resolved by 10% SDS-PAGE and transferred to nitrocellulose membranes. The membrane was blocked with 5% non-fat milk at room temperature for 1 h and then incubated with primary antibodies at 4 °C overnight. The blots were developed with ECL Prime reagents from GE Life Sciences (Piscataway, NJ, USA).

### 3.7. Data and Statistics

Microarray data were tested with Benjamini and Hochberg False Discovery Rate multiple testing correction. Other data were presented as mean ± SEM and tested with unpaired Student’s *t*-test or one-way ANOVA as appropriate, with a value of *p* < 0.05 being regarded as statistically significant. SPSS18.0 was used for statistical analysis.

## 4. Conclusions

In conclusion, to our knowledge this is the first genome-wide characterization of the gene expression profile in response to Sirt2 knockdown in endothelial cells. Sirt2-sensitive genes are involved in multiple cellular functions. Pharmacological inhibition of Sirt2 attenuated oxidant-induced endothelial cell death. These data suggest that Sirt2 is functionally important in endothelial cells under oxidative stress. Our results may provide a basis for future studies aiming to dissect the specific signaling pathway(s) that mediates specific Sirt2 functions in endothelial cells. Nevertheless, a limitation of the present study was that the microarray data did not provide direct evidence about the specific gene products that were involved in mediating the observed effects of Sirt2. Given the number of genes that are responsive to the changed Sirt2 level, it is likely that multiple mechanisms may be implicated in each specific biological function of Sirt2.

## Figures and Tables

**Table 1:** List of differentially expressed genes after Sirt1 or Sirt2 gene silencing (read the entire table column-wise).

Sirt1		
11716338_a_at	U	INSIG1
11716395_a_at	U	GPR56
11718915_a_at	U	RGS19
11719218_at	U	SOCS3
11719513_a_at	U	ADAM15
11719745_s_at	U	ARHGAP27
11720832_x_at	U	SOX18
11722324_a_at	U	ZNF84
11722353_s_at	U	LDB2
11724390_x_at	U	ZDHHC16
11724394_at	U	C2orf55
11724395_a_at	U	ZNF83
11724396_x_at	U	ZNF83
11727984_at	U	ODZ3
11728347_at	U	ABI3
11729918_at	U	ADAMTS18
11729919_a_at	U	ADAMTS18
11730211_x_at	U	PFKL
11731622_x_at	U	ZNF91
11733299_a_at	U	CLDN5
11736013_at	U	GLE1
11736029_a_at	U	ITGA6
11736458_x_at	U	KCNK6
11737089_a_at	U	TLL1
11737870_s_at	U	FAM78A
11739146_a_at	U	MKNK2
11739491_a_at	U	MGAT4A
11739492_a_at	U	MGAT4A
11739493_at	U	MGAT4A
11739650_at	U	DLL4
11740601_a_at	U	APH1B
11722277_s_at	U	ZBED4
11722388_at	U	MTF1
11722391_at	U	MTF1
11722531_a_at	U	CALD1
11722532_s_at	U	CALD1
11722533_x_at	U	CALD1
11723092_at	U	FNIP2
11723414_a_at	U	CNN2
11723416_x_at	U	CNN2
11724389_a_at	U	ZDHHC16
11725054_a_at	U	HNMT
11726328_x_at	U	GBP1
11726796_a_at	U	NEXN
11726797_x_at	U	NEXN
11726824_a_at	U	ZNF565
11727545_at	U	PANK3
11727784_x_at	U	TPM4
11728028_a_at	U	PWWP2A
11728226_a_at	U	CASP7
11728276_s_at	U	PLA2G12A
11728488_a_at	U	NRXN3
11728744_at	U	C4orf46
11729665_a_at	U	STK38L
11730613_at	U	MBOAT7
11731665_a_at	U	PDGFB
11732339_at	U	BCL11A
11733043_a_at	U	SLC7A2
11733084_a_at	U	PALLD
11734281_a_at	U	ZNF17
11734865_a_at	U	PSMC3IP
11736812_at	U	PDCD1LG2
11736959_a_at	U	TNFSF15
11724786_s_at	D	C14orf129
11725510_a_at	D	SLC7A6
11725569_at	D	KLHL15
11725596_a_at	D	ZDHHC23
11725733_a_at	D	NFYA
11725850_at	D	USP48
11726455_x_at	D	MYO5B
11726551_s_at	D	RAET1L
11726552_x_at	D	ULBP2
11727222_at	D	EVI5
11727286_a_at	D	ZNF323
11727361_a_at	D	MYLK
11727406_a_at	D	TEK
11727485_at	D	TPR
11727854_s_at	D	NUP50
11727905_a_at	D	IL13RA1
11727984_at	D	ODZ3
11728195_s_at	D	TRO
11728288_a_at	D	KRT15
11728353_at	D	MSMP
11728960_a_at	D	KRT80
11729449_s_at	D	TINF2
11729550_a_at	D	GPR126
11729840_s_at	D	ZCCHC2
11730033_a_at	D	RPS6KL1
11730195_at	D	SEC14L2
11730615_a_at	D	PLCD4
11730616_at	D	PLCD4
11730623_at	D	DSEL
11732160_a_at	D	NUDCD1
11732713_at	D	FST
11733051_a_at	D	SCAMP1
11740602_s_at	U	APH1B
11740624_a_at	U	AZI1
11742830_a_at	U	PHF3
11744239_a_at	U	CCDC159
11744430_a_at	U	KIF9
11744505_x_at	U	TP53I11
11744948_x_at	U	SEMA3F
11745450_a_at	U	AUH
11745927_x_at	U	ADAM15
11746516_a_at	U	ADAM15
11747060_a_at	U	MGAT4A
11747977_a_at	U	ZNF180
11748731_a_at	U	ZNF616
11749511_a_at	U	ZNF595
11751946_a_at	U	ARHGAP21
11752002_a_at	U	PLEKHG1
11752675_a_at	U	OGFR
11754022_s_at	U	GAP43
11754446_x_at	U	ZNF761
11754754_s_at	U	ADAMTS18
11755219_a_at	U	THBD
11755232_s_at	U	ZNF468
11755474_a_at	U	ADAM15
11757861_a_at	U	TSHZ1
11760814_x_at	U	KANK3
11715679_s_at	D	PRNP
11715889_a_at	D	ATP5I
11715890_x_at	D	ATP5I
11716027_at	D	SELT
11716028_x_at	D	SELT
11716404_s_at	D	EIF4EBP2
11718102_at	D	CD59
11718141_at	D	DKK1
11718769_a_at	D	MAPKSP1
11719164_a_at	D	CLCN5
11719267_s_at	D	SERINC1
11719394_a_at	D	FBXO32
11719395_at	D	EXTL2
11719396_a_at	D	EXTL2
11719479_at	D	ALOX5AP
11719712_at	D	PPM1B
11719816_s_at	D	BET1
11720240_at	D	TMSB15A
11720514_at	D	C9orf150
11721024_a_at	D	IL11RA
11721112_a_at	D	ACP6
11739010_a_at	U	MYH9
11739086_x_at	U	MESDC2
11739088_at	U	MESDC2
11739451_a_at	U	FRMD6
11739672_x_at	U	ZNF253
11740133_a_at	U	CALD1
11740743_a_at	U	TPM2
11740744_x_at	U	TPM2
11741168_a_at	U	MSRB3
11741188_a_at	U	SLC30A6
11742483_a_at	U	C1orf110
11743015_a_at	U	DIP2C
11743020_at	U	ZSCAN29
11743253_x_at	U	CALD1
11743458_a_at	U	FAM49A
11743696_at	U	CLEC14A
11743705_at	U	ETAA1
11744034_a_at	U	VASP
11744323_s_at	U	PWWP2A
11745608_a_at	U	WDR1
11745924_at	U	LOC220930
11746173_a_at	U	DDHD1
11746476_x_at	U	CALD1
11746548_s_at	U	CNN2
11747300_a_at	U	CDK14
11747469_x_at	U	KDM2B
11747711_a_at	U	LDB1
11748208_a_at	U	ZNF317
11748391_x_at	U	ZDHHC16
11748400_s_at	U	LOC643634
11748401_x_at	U	TPM4
11748403_x_at	U	CNN2
11748527_a_at	U	ARHGAP24
11749172_x_at	U	NEXN
11749732_a_at	U	LONP2
11749734_s_at	U	JRKL
11749921_a_at	U	SDC4
11750198_a_at	U	CASP7
11750623_a_at	U	FILIP1L
11751244_s_at	U	CNN2
11751245_x_at	U	CNN2
11751993_a_at	U	JAG1
11752164_x_at	U	KDM2B
11752276_a_at	U	DIDO1
11752361_s_at	U	NEXN
11752499_a_at	U	CALD1
11733054_a_at	D	SCAMP1
11733929_a_at	D	ARMC8
11734059_a_at	D	PSTK
11734150_x_at	D	PLAGL1
11735224_a_at	D	KLRG1
11735991_at	D	LARS
11736192_at	D	RRM2B
11736343_x_at	D	OPA3
11736345_x_at	D	OPA3
11736528_a_at	D	SMC2
11736785_at	D	HOXA2
11737052_x_at	D	PLAGL1
11737816_x_at	D	FAM119B
11739064_s_at	D	GNG12
11739245_a_at	D	ANKFY1
11739596_a_at	D	KIAA1429
11739640_at	D	DIP2A
11739942_s_at	D	SEPSECS
11740096_a_at	D	TMCC1
11740176_at	D	ARSK
11740213_a_at	D	TBL1X
11741152_x_at	D	PLAGL1
11742720_at	D	LRRC58
11742722_at	D	LRRC58
11742962_a_at	D	IP6K2
11743404_at	D	ZMAT2
11743573_at	D	TMEM184C
11743574_x_at	D	TMEM184C
11743648_a_at	D	DCAF6
11743649_a_at	D	DCAF6
11743763_at	D	GTF3C3
11744083_at	D	ANKIB1
11744415_s_at	D	MFSD6
11744788_x_at	D	TMEM68
11745010_a_at	D	DCBLD2
11745230_a_at	D	C3orf23
11745231_a_at	D	C3orf23
11745700_s_at	D	ULBP2
11746163_a_at	D	LARP4B
11746536_a_at	D	WSB2
11747146_s_at	D	TMBIM6
11748416_a_at	D	DCAF6
11749027_x_at	D	HERPUD2
11750354_a_at	D	TMEM184C
11750993_x_at	D	MAP3K7
11751165_a_at	D	RBMS2
11722843_a_at	D	ENAH
11723533_x_at	D	BRMS1L
11723534_at	D	BRMS1L
11723580_at	D	LOC221710
11724238_at	D	CYB5R4
11726140_s_at	D	SIRT1
11726750_a_at	D	GTF3C4
11727022_at	D	TMEM64
11727370_at	D	TSN
11727935_at	D	C4orf49
11729128_at	D	CPA4
11729710_a_at	D	MARS2
11731195_at	D	SEMA3D
11731263_a_at	D	ZNF365
11734371_a_at	D	SCML1
11734955_a_at	D	SCML1
11736470_at	D	SLC35D1
11738893_s_at	D	TPM1
11739119_s_at	D	CNPY3
11742308_s_at	D	TPM1
11742483_a_at	D	C1orf110
11742743_a_at	D	CNN3
11743092_at	D	THEM4
11743197_at	D	TLR4
11743334_a_at	D	MRPL35
11743973_a_at	D	MRPL38
11743974_at	D	MRPL38
11745482_s_at	D	PRNP
11746622_a_at	D	PHC1
11746928_a_at	D	ENC1
11747834_a_at	D	SPATA5
11748315_s_at	D	PRNP
11750985_a_at	D	EXTL2
11751191_a_at	D	LIPG
11752333_a_at	D	ITGAV
11754940_s_at	D	TSN
11754976_x_at	D	CNPY3
11755458_a_at	D	HDDC2
11755848_a_at	D	C17orf51
11756471_a_at	D	MFSD2A
11756882_a_at	D	RTTN
11757738_s_at	D	FAT1
11758013_s_at	D	C8orf4
11758101_s_at	D	EIF4EBP2
11758326_s_at	D	THEM4
11758872_at	D	CDC37L1
11752930_a_at	U	GBP1
11754084_x_at	U	MYL9
11754644_x_at	U	CNN2
11754887_a_at	U	MSRB3
11754911_x_at	U	NEXN
11755122_a_at	U	PALLD
11755734_x_at	U	CCDC107
11757637_a_at	U	MUS81
11758013_s_at	U	C8orf4
11759169_a_at	U	C1orf222
11759711_a_at	U	RBM25
11760202_at	U	IGFBP7
11760611_x_at	U	SETD6
11760918_a_at	U	MRRF
11763500_a_at	U	ZNF93
11715207_at	D	WDFY4
11715265_at	D	FIG4
11715477_at	D	TFRC
11715545_at	D	TMED10
11715550_at	D	DAG1
11715651_s_at	D	FSTL1
11715761_a_at	D	TBC1D14
11716015_a_at	D	CMTM3
11716208_s_at	D	GLUD1
11716288_s_at	D	ESYT1
11716391_a_at	D	BACE1
11716620_a_at	D	TNPO1
11716626_at	D	KIF3B
11716787_a_at	D	B3GNT1
11716788_at	D	B3GNT1
11718184_a_at	D	FCHSD2
11718287_at	D	UBL3
11718288_at	D	UBL3
11718406_s_at	D	TMBIM6
11718439_at	D	NSUN4
11718734_a_at	D	POGK
11718900_a_at	D	TGFBR3
11718901_at	D	TGFBR3
11719088_at	D	MMGT1
11719353_s_at	D	GCC2
11719397_a_at	D	RRAGC
11719398_s_at	D	RRAGC
11719409_a_at	D	HIPK2
11719628_a_at	D	HDHD1A
11719786_at	D	SMCR7L
11719912_a_at	D	KDM1B
11751297_s_at	D	SUB1
11751305_a_at	D	B3GNT1
11751353_a_at	D	RRAGC
11751354_a_at	D	HDHD1A
11753308_s_at	D	ULBP2
11753549_a_at	D	CMTM3
11754031_s_at	D	CKS1B
11754827_x_at	D	FBXO17
11755251_x_at	D	FADS1
11756152_s_at	D	PCNP
11756156_s_at	D	TFRC
11756181_x_at	D	YWHAZ
11756205_x_at	D	DCAF6
11756254_a_at	D	GGT5
11756259_s_at	D	NFATC1
11756285_s_at	D	IGF2BP3
11756497_a_at	D	VWCE
11756603_a_at	D	C9orf6
11756861_s_at	D	ULBP2
11757430_s_at	D	TMED5
11757523_s_at	D	WDR35
11757542_s_at	D	SSR1
11757787_x_at	D	FTL
11757799_s_at	D	VAMP7
11757810_s_at	D	TMED10
11757880_s_at	D	DAG1
11757989_s_at	D	ANKRD46
11758200_x_at	D	CKS1B
11758212_s_at	D	KIAA0494
11758452_s_at	D	CENPQ
11758454_s_at	D	FAM116A
11758750_x_at	D	YWHAZ
11758751_at	D	YWHAZ
11758809_at	D	RRAGC
11758820_at	D	DNAJC10
11758873_a_at	D	HPSE
11758995_at	D	LOX
11758999_s_at	D	UBE2H
11759720_x_at	D	LOC400590
11763276_a_at	D	PWP1
11763395_a_at	D	ZC3HAV1L
11764275_s_at	D	SLC11A2
**Co-regulated**		
11715698_a_at	U	NOLC1
11717961_at	U	MED11
11718908_s_at	U	CHST2
11758929_at	D	TFDP2
11759503_at	D	FAM103A1
11759776_at	D	GTDC1
11759943_at	D	FAM13A
**Sirt2**		
11715270_s_at	U	KLF7
11715586_at	U	MAPRE1
11715713_a_at	U	VOPP1
11715803_a_at	U	ANXA6
11716299_a_at	U	ITGAV
11716344_a_at	U	ZCCHC3
11716413_x_at	U	MT1E
11716582_a_at	U	G3BP2
11716771_s_at	U	SIK1
11717055_a_at	U	CORO1C
11717056_a_at	U	CORO1C
11717952_at	U	ZDHHC18
11717968_at	U	TMEM216
11719647_a_at	U	CASP7
11719648_a_at	U	CASP7
11719833_at	U	MPZL2
11720028_x_at	U	LDLR
11720063_a_at	U	GLIPR2
11720223_at	U	GFPT1
11720823_at	U	JAG1
11720849_a_at	U	RAB23
11721218_a_at	U	MSRB3
11721525_s_at	U	LOC440354
11721684_a_at	U	ZW10
11721778_a_at	U	ACBD5
11722193_a_at	U	C12orf75
11722218_a_at	U	WBP4
11722220_a_at	U	WBP4
11720046_x_at	D	DNAJC10
11720111_at	D	SNTB2
11720112_at	D	SNTB2
11720146_a_at	D	DAPK1
11720273_at	D	SFT2D3
11720570_a_at	D	PHF15
11720599_s_at	D	SUB1
11720602_at	D	SYT11
11720798_at	D	RAB8B
11720799_s_at	D	RAB8B
11720800_a_at	D	RAB8B
11720893_s_at	D	SOS1
11721524_s_at	D	ZNF706
11721585_a_at	D	TMCC1
11721622_a_at	D	KATNAL1
11721834_a_at	D	GET4
11721993_at	D	SLC6A6
11722111_at	D	HLX
11722273_s_at	D	KIAA1826
11722338_at	D	PEX7
11722377_at	D	PNPO
11722425_s_at	D	NEDD4L
11722460_at	D	PCDH12
11722475_a_at	D	ARID3B
11722662_a_at	D	HPSE
11722969_s_at	D	TRPV4
11722977_at	D	HOXB5
11723228_s_at	D	SGCB
11723230_a_at	D	RNF138
11723586_a_at	D	GRB14
11723639_s_at	D	C11orf58
11724011_at	D	SIKE1
11724171_a_at	D	FCHO2
11718909_x_at	U	CHST2
11721860_s_at	U	STX12
11721862_a_at	U	STX12
11722853_a_at	U	HABP4
11724388_at	U	ZNF721
11724959_s_at	U	CDK14
11727522_a_at	U	ZNF267
11727523_x_at	U	ZNF267
11727782_a_at	U	TPM4
11727783_s_at	U	TPM4
11728155_s_at	U	FUT4
11731868_a_at	U	BICD2
11734554_a_at	U	BCL7B
11738988_a_at	U	GANAB
11738989_a_at	U	GANAB
11738990_x_at	U	GANAB
11743449_a_at	U	BICD2
11744786_x_at	U	OGFR
11748926_a_at	U	GANAB
11752163_a_at	U	KDM2B
11752676_x_at	U	OGFR
11753089_a_at	U	OGFR
11753090_x_at	U	OGFR
11754869_s_at	U	ZNF267
11755270_a_at	U	GANAB
11757525_s_at	U	BICD2
11759004_at	U	SLC33A1
11759757_a_at	U	SLC33A1
11741758_x_at	D	TRPV4
11754398_at	D	LOC644538
11758147_s_at	D	MAPK13
11758902_at	D	ZNF641

## Supplementary Files

Supplementary File 1 supplementary file (TIFF, 2961 KB)
