# Supplementary figures and images for: Global Gene Expression Profiling Reveals Functional Importance of Sirt2 in Endothelial Cells under Oxidative Stress
Source: Int J Mol Sci. 2013 Mar 11;14(3):5633–49. doi: 10.3390/ijms14035633 (PMC3634502; doi:10.3390/ijms14035633)

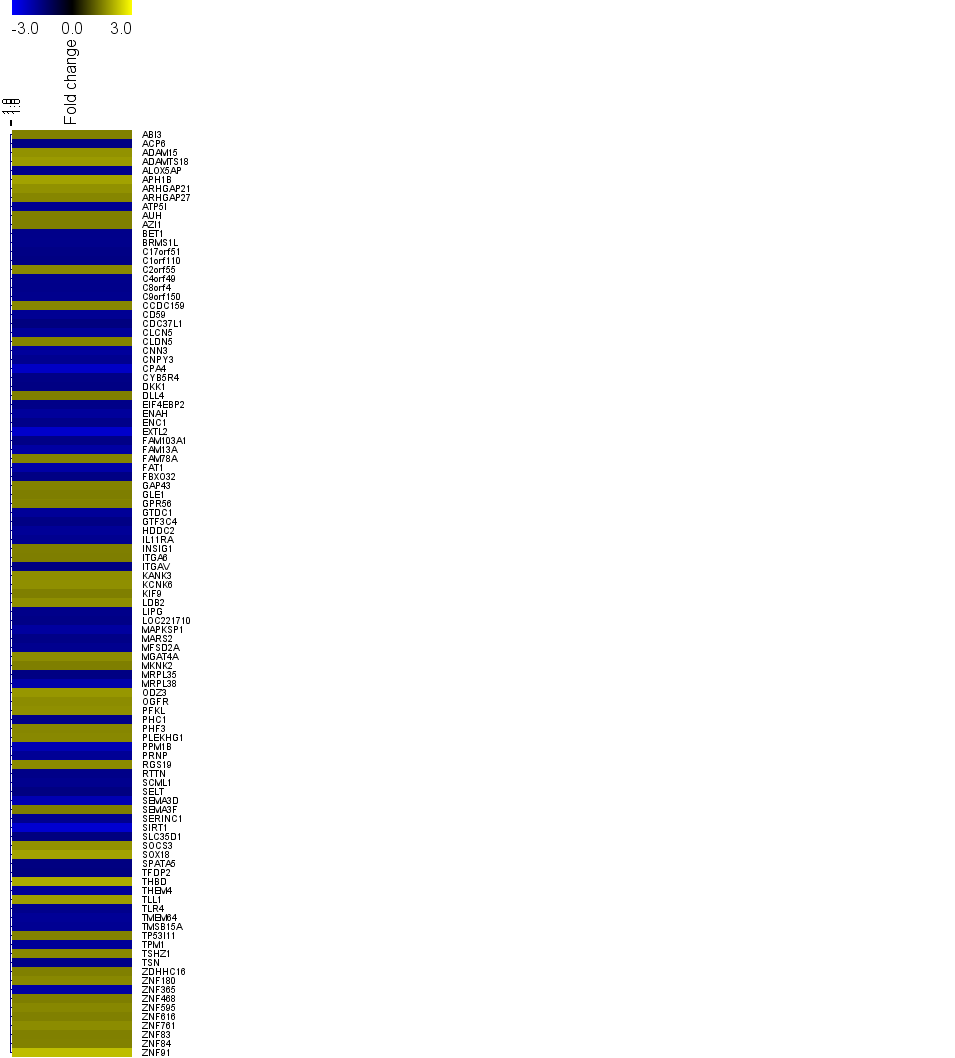

Supplement: Supplementary File 1 — supplementary file (TIFF, 2961 KB) [file ijms-14-05633-s001.tiff]
